# A hidden Markov model-based algorithm for identifying tumour subtype using array CGH data

**DOI:** 10.1186/1471-2164-12-S5-S10

**Published:** 2011-12-23

**Authors:** Ke Zhang, Yi Yang, Viswanath Devanarayan, Linglin Xie, Youping Deng, Sens Donald

**Affiliations:** 1 Department of Pathology, School of Medicine and Health Sciences, University of North Dakota, Grand Forks, ND 58201, USA; 2 Exploratory Statistics, Global Pharmaceutical Research & Development, Abbott Laboratories, Abbott Park, IL 60064, USA; 3 Department of Biochemistry and Molecular Biology, School of Medicine and Health Sciences, University of North Dakota, Grand Forks, ND 58201, USA; 4 Department of Internal Medicine, Rush University Medical Center, Chicago, IL 60612, USA

## Abstract

**Background:**

The recent advancement in array CGH (aCGH) research has significantly improved tumor identification using DNA copy number data. A number of unsupervised learning methods have been proposed for clustering aCGH samples. Two of the major challenges for developing aCGH sample clustering are the high spatial correlation between aCGH markers and the low computing efficiency. A mixture hidden Markov model based algorithm was developed to address these two challenges.

**Results:**

The hidden Markov model (HMM) was used to model the spatial correlation between aCGH markers. A fast clustering algorithm was implemented and real data analysis on glioma aCGH data has shown that it converges to the optimal cluster rapidly and the computation time is proportional to the sample size. Simulation results showed that this HMM based clustering (HMMC) method has a substantially lower error rate than NMF clustering. The HMMC results for glioma data were significantly associated with clinical outcomes.

**Conclusions:**

We have developed a fast clustering algorithm to identify tumor subtypes based on DNA copy number aberrations. The performance of the proposed HMMC method has been evaluated using both simulated and real aCGH data. The software for HMMC in both R and C++ is available in ND INBRE website http://ndinbre.org/programs/bioinformatics.php.

## Background

Tumor progression is a complicated biological process that comes with enormous genetic and molecular changes, such as chromosome aberration, gene mutations, and activation or inhibition of transcriptional pathways. The abnormal genetic changes often show high variability even among tumors within the same histopathological subtype and anatomical origin, which may lead to variation in clinical outcomes. For example, a subtype of colorectal cancer, hereditary nonpolyposis (HNPCC), is characterized by dominant genetic defects in DNA mismatch repair pathway and HNPCC patients have higher 5-year survival than other subtypes of colorectal cancer patients[1]. When genetic aberration is specific to a subset of tumors, it provides potent targets for chemotherapy. Examples include trastuzumab and lapatinib for treating HER2-positive breast cancers [2], tamoxifen for treating ER-positive breast cancers[3,4], and gefitinib and erlotinib for non-small cell lung cancer with EGFR mutations [5-9].

DNA copy number aberration is a striking feature of tumor cell. During tumor progression, chromosome is subjected to dramatic change in that DNA segments are amplified, deleted, or translocated. Comparative genomic hybridization (CGH) technology has been a widely used tool for detecting changes in chromosome fragments. The advancement of array technology has enabled researchers to conduct array CGH (aCGH) study for profiling genome-wide chromosome variations using high density array, such as single nucleotide polymorphism (SNP) array that contains from 100K to 3M SNP markers [10]. Such high dimensional DNA copy number data reveals genomic heterogeneity in many cancer types, ensuring biomarker discovery for each genomic subtype at SNP copy number level [11].

Multiple clustering methods, including hierarchical clustering (HC), Naive Bayes, K-nearest neighbours, support vector machine, probability model-based clustering, and nonnegative matrix factorization (NMF), have been developed and applied for aCGH data to identify tumor subtypes based on the DNA copy number aberrations [12-15]. We have previously developed a revised version of NMF that showed improved performance for aCGH clustering when testing on three tumor types, non-small cell lung carcinoma, colorectal cancer and malignant melanoma [16]. However, all these aforementioned methods fail to account for the spatial correlation between SNPs, and the correlation between adjunct SNPs could be as high as 0.99 for high density SNP array such as Affymetrix 500K. We therefore developed a mixture model based clustering method for tumor subtype classification that uses hidden Markov model (HMM) to account for the spatial correlation in aCGH data.

Shah *et al. *[17] have proposed a similar HMM based clustering method to account for spatial correlation, but is fundamentally different from our clustering method in a number of ways First, the models are different. They proposed a Bayesian hierarchical model, nonetheless we fit a hidden Markov model to the data directly, therefore it has less unknown variables and decreases the risk of model overfitting. Secondly, they use expectation maximization (EM) like algorithm to estimate variables, and we use maximum likelihood based method that is computationally more efficient. Thirdly, our algorithm automatically finds the optimal number of groups and converges to the optimal grouping. Fourthly, we developed a machine learning clustering algorithm and have implemented it using C++ parallel programming for fast computation. Finally, we pre-process the raw aCGH data by segmenting the chromosome prior to clustering. The segmentation step normalizes aCGH data that usually contains high frequency of intensity noise. We show the performance of the proposed HMM-based clustering (HMMC) method by applying it to simulated data and aCGH glioma data.

## Methods

We first describe the data pre-processing procedure, our HMM model and the model fitting for a cluster of aCGH samples. Then we describe the machine learning algorithm that uses HMM models to cluster tumors. Finally, we introduce our fast implementation for the clustering algorithm and the approach to find the optimal number of groups.

### aCGH data segmentation

Raw data from Affymetrix SNP array was preprocessed using the open source R package aroma.affymetrix with the default parameters [18]. The resulting SNP-level copy numbers were segmented using circular binary segmentation (CBS) method within aroma.affymetrix [19]. The break points predicted by CBS at each sample were then summarized across all samples and the segments between pairs of adjunct break points were identified and the mean copy number of each segment was calculated for clustering analysis. Because tumor specimen is often contaminated with normal tissues that are adjacent to them, which would complicate the analysis and introduce false negatives, it is necessary to conduct quality control to remove the normal contamination first. We developed a machine learning algorithm to identify and filter out contaminated samples based on segmented copy number data. To conduct the quality control, we first chose two groups of samples; one with the highest number of copy number alteration regions and the other being normal samples. These two groups of samples served as a training dataset for a Random Forest classifier [20] that was used to distinguish tumors and normal samples and identify signatures for tumor samples. The out of bag error rate of this classifier is usually very low (<5%). The trained classifier was then applied to all aCGH samples and a score of probability to be contaminated was calculated for each sample. Samples with a probability of more than 50% of contamination were excluded from further clustering analysis.

### HMM model fitting

**Figure 1:**
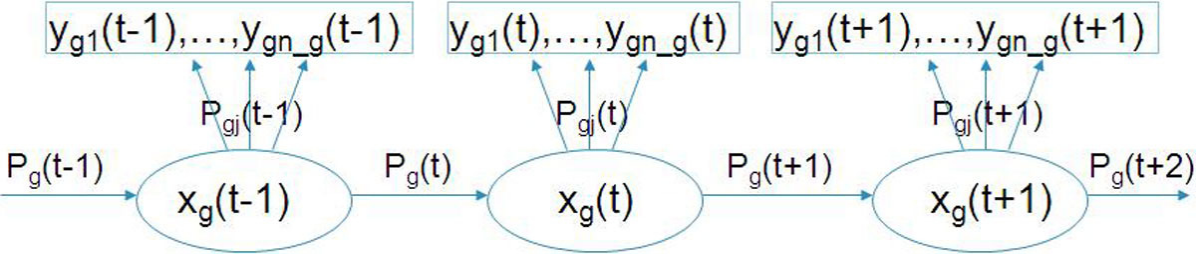
**A hidden Markov model for a cluster of tumor aCGH samples**. There are n_g samples in cluster g, and the graph shows three DNA segment, t-1, t, and t+1. X is the unobserved copy number for each segment and y is the observed mean intensity value for each sample. P_g _is transitional probability and P_gj _is emission probability.

We use a mixture HMM model to represent the clusters of tumor samples in that each cluster or subtype of tumors is modelled by a single HMM. Let t = 1,.., T, denote the DNA segments of aCGH data. Suppose there are G clusters in total and there are n_g _samples in cluster g, where g = 1,..., G. The HMM model for cluster g is demonstrated in Figure 1. The hidden state at segment t is denoted as x_g_(t), which is the true copy number of segment t and is unknown. The observed mean copy number for segment t and sample j is denoted as y_gj_(t) where j = 1, ..., n_g_. The transitional probability from state t-1 to t is denoted as P_g_(t) and the emission probability from state t to observation y_gj_(t) is denoted as P_gj_(t).

Based on the distribution of segmentation copy number data, we choose 6 states, xX{0.1, 1.5, 2, 3, 4, 5}. The transitional probabilities between states are given in a multinomial distribution that was empirically estimated from the raw data. For example, for the 79 glioma samples we discuss in the results section, the transitional probability between the same states is 0.56, and is 0.088 otherwise. Lognormal distribution is widely used to model DNA copy number [21]. We let the emission probability follow a log-normal distribution with the hidden state value being the mean of the normal distribution. The standard deviation of the normal distribution was empirically estimated from raw data using the mean standard deviation of segment copy number across all samples. Viterbi algorithm is used to fit this HMM model to a cluster of tumor samples. Viterbi algorithm is an efficient algorithm that uses a dynamic programming approach to estimate the hidden states from segment 1 to T and to calculate delta value, Δ_g_, which is the log-likelihood for the model fitting of cluster g. We have implemented Viterbi algorithm in both R and C++ programming environment.

### Sample clustering

The optimal clustering is determined by the maximum sum of delta values from all groups. It is computationally infeasible to perform HMM fitting and calculate delta sum values for every possible clustering because the number of clustering for n samples G groups is G^n^. We have developed a computationally efficient algorithm for finding the clustering optima.

**Figure 2:**
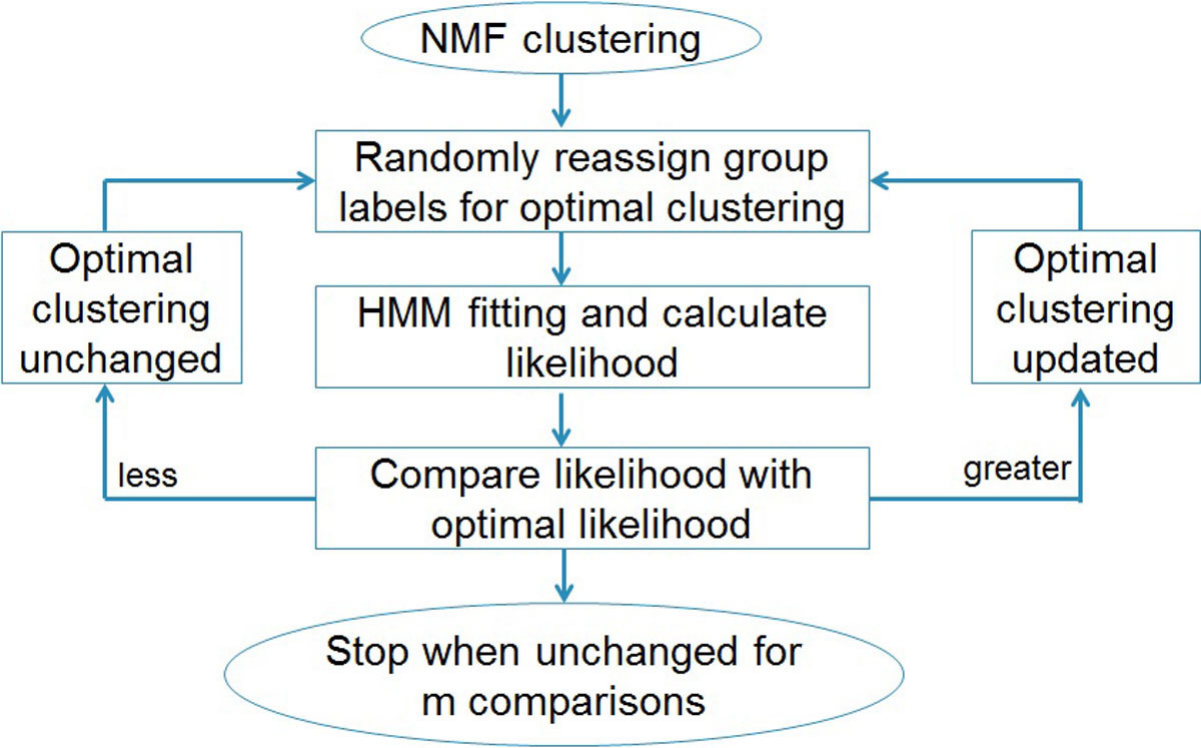
**Workflow of HMMC clustering procedure**. The procedure stops when the optimal clustering is not updated for m = n^2 ^comparisons where n is the number of samples.

The algorithm searches the optimal clustering as follows. First, for a given number of groups G, we run a NMF algorithm that was developed earlier to preliminarily cluster the samples. The NMF algorithm has been described previously [16]. Though NMF classifies aCGH data without considering the correlation between markers, it has been shown to discover genomic signatures in various types of tumors [11,16]. Thus, the clustering result of NMF serves as a starting point for HMM clustering. Secondly, we fit a HMM for each cluster from the previous step and calculate the sum of delta values, Δ_1_, ..., Δ_G_. This is the log-likelihood for the current optimal clustering. Thirdly, based on the current optimal clustering, we randomly select 2 samples, and re-assign the group labels to them. We then perform HMM model fitting and calculate the log-likelihood for the new clustering. If the new log-likelihood is greater than the optimal log-likelihood, we update the optimal clustering with this new clustering. Fourthly and finally, we repeat the last step a number of times, and stop until the optimal log-likelihood remains unchanged for m times of consecutive random clustering. We find that m = n^2 ^is sufficient for HMMC to converge to the optimal clustering. This workflow of clustering procedure is illustrated in Figure 2.

We have use both R and C++ to implement this clustering algorithm. As step 3 can be performed simultaneously for multiple random clustering, we implemented a parallel computing version of HMMC to decrease the computation time. This was accomplished using the OpenMP package [22] within C++ to allow the random clustering and HMM fitting to be carried out across multiple threads in a multi-core machine. Each thread can update the optimal clustering and all threads share the same optima. We have tested this program in Dell PowerEdge R910 workstation that has 32-cores and the parallel computing increased efficiency by an order of magnitude.

### Model selection

One of the most difficult questions about clustering problem is to determine the number of groups, G. Our HMMC algorithm relies on a given G to find the optimal clustering. Thus model selection is needed to determine the best number of groups.

We use the Bayesian Information Criterion (BIC), a widespread statistical tool to conduct model selection. BIC is defined as follows:

BIC=-2lnL+k× ln(n),

where *L *is the likelihood which measures how good the HMM model approximate the data, *k *is the number of parameters used in the model, and *n *is the number of samples. The second term, *k*ln*(n)*, serves as a penalty on the number of parameters used in the model to avoid overfitting. Thus, the magnitude of penalty increases as the number G increases. A preferred model is selected upon a lower BIC value that indicates relatively higher likelihood as well as relatively lower risk of overfitting.

The sum of delta values is the log-likelihood for HMM model fitting,

$$\ln L=\overset{G}{\underset{g=1}{\sum }}\Delta_{g},$$

where **Δ**g is the delta value of the *g*th group. Because the parameters for transitional and emission probability are empirically estimated, the number of unknown parameters k in BIC is equal to the number of hidden states. For an aCGH dataset with G groups and T segments, the number of parameters is equal to T × G. Thus, the BIC for HMM is defined as

$$BIC=-2\overset{G}{\underset{g=1}{\sum }}\Delta_{g}+T\times G\times \ln\left(n\right).$$

We run HMMC for a range of values of G, and select the optimal G with the smallest BIC.

### Cross-validation for testing stability of HMMC

To assess the stability of HMMC results, we conduct a cross-validation as described earlier [16]. After completing HMMC for the entire dataset, we perform cross-validation as follows. We randomly leave 10% of samples out and apply HMMC to the remaining 90% of samples and compare the clustering results with the original one. The number of samples that are assigned to a different subgroup other than the original result is counted as errors. We repeat this procedure 100 times and calculate the error rate, which represents the stability of the clustering algorithm with respect to the permutation of samples.

## Results

### Simulation study

In order to test the performance of HMMC, we conducted simulation study to compare HMMC with NMF clustering. We created two groups of aCGH data with 10 samples per group and 100 segments per each sample. For the first group, all data were generated from a normal distribution with mean equal to 2 and standard deviation equal to s, which was variable between different experiments. For the second group, the copy number of all segments followed a normal distribution as well. This normal distribution was the same as the first group, except that 3 segments in the middle of genome had a mean equal to 4 such that these 3 segments were amplified. In order to introduce random noise, we randomly drew 40 segments in all samples and increased their copy number values by 2 (their mean was equal to 4). Thus these 40 segments were randomly amplified segments. Furthermore, AR(1) correlation was introduced into the data. Considering the high correlation between SNPs in the high density SNP array, we set the pho value of AR(1) to be 0.9.

**Figure 3:**
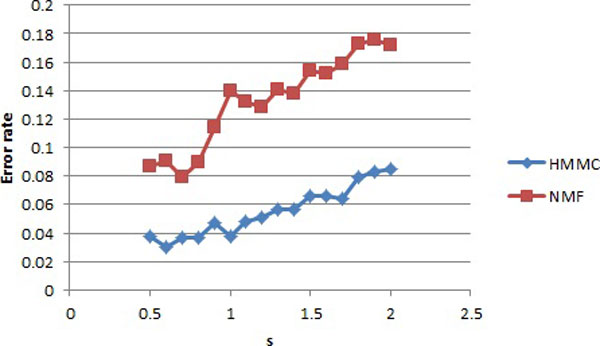
**The error rates for simulated data**. HMMC and NMF were tested on the same simulated data. The x axis s is the standard deviation that was used to generate the data. The blue curve is the error rates of HMMC, and the red curve is for NMF.

We used HMMC and NMF to cluster the two groups with s value varying from 0.5 to 2. For each s, 200 aCGH datasets were generated and the number of samples misplaced for either method was counted. The error rate curves are shown in Figure 3.

The error rates of both HMMC and NMF increase as the standard deviation s increases. The error rates of HMMC are in the range of 3% to 8.5%, whereas those for NMF are between 7.9% and 17.6%. Therefore, the chance for HMMC to incorrectly classifying samples is less than half of that for NMF in our simulation study.

### Glioma data

Glioma tissue aCGH data were acquired from Repository for Molecular BRAin Neoplasma DaTa (REMBRANDT) [23]. We obtained Affymetrix 500K SNP array data and the corresponding clinical information for 79 glioma patients. The aCGH data was first processed using aroma.affymetrix software and was then segmented using circular binary segmentation method. This preprocessing step reduced the number of markers from 500K to 5321 segments. The segmented data was used for further analysis.

**Figure 4:**
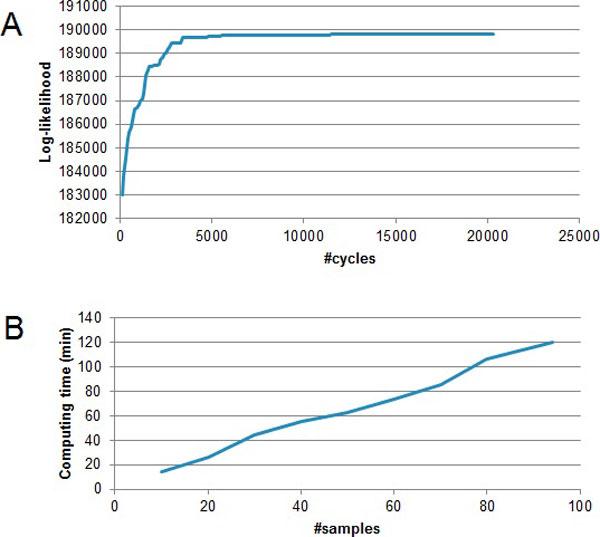
**Computational performance for glioma data**. A. The log-likelihood of HMMC converges rapidly to its maximum. B. The computational time of HMMC is linear to the sample size.

The first question to be addressed is the computational performance of HMMC when it is applied to high-dimensional data. We first test the convergence rate of HMMC for glioma aCGH data assuming 2 groups. As shown in Figure 4A, the log-likelihood of HMMC increases rapidly in the first 4,000 cycles of clustering, and it remains stable after 5,000 cycles. The log-likelihood reaches its maximum at approximately 15,000 cycles, and the computation stops after an additional 79^2 ^= 6241 cycles.

Secondly, we tested the computation time for different number of samples. Random samples were drawn from the 79 glioma data. The numbers of random samples was from 10 to 95 and the number of groups was assumed to be 2. We used a Dell PowerEdge R910 to run 20 threads simultaneously for this test. Figure 4B shows the curve of computation time. For 10 samples, it took less than 20 minutes. For 95 samples, it took about 2 hours to find the optimal clustering. The computation time is approximately linear to the number of samples (*O*(n)). Therefore, our clustering algorithm is computationally efficient and it achieves *O*(n) for the computational complexity.

To find the optimal number of groups, we conducted model selection using BIC. We ran HMMC for the number of groups G from 2 to 6, and calculated the corresponding maximum log-likelihood and BIC. The results are shown in table 1. The maximum log-likelihood increases as the number of groups increases because the model has a better fit for the data as the number of parameters increases. The BIC value was -304,617 for 2 groups, then it decreased to -305,030 for 3 groups, and then increased as the number of groups increased. Thus, the optimal number of groups is 3 because it gave the minimum value of BIC.

**Table 1:** Model selection for glioma data

#Group	2	3	4	5	6
Maximum log-likelihood	162406	167661	169978	172476	175554

BIC	-304617	-305030	-299567	-294466	-290524

**Figure 5:**
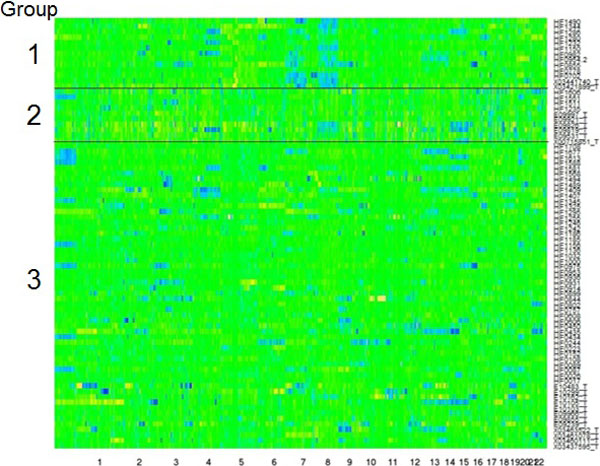
**Heatmap of glioma clustering**. 79 glioma samples with 5,321 segments were clustered into 3 groups using HMMC. Sample names are labelled on the right side, and chromosome number is marked at the bottom. Yellow denotes amplification and blue denotes deletion. The three groups are labelled with number 1 to 3 on the left side of the graph.

Figure 5 shows the heatmap of clustering the glioma samples into 3 groups. Each row of the heatmap is a glioma sample, and each column is a DNA segment. There are 79 rows and 5,321 columns totally. The segments are sorted in the order of genomic positions from chromosome 1 to 22. Sex chromosome was not included to avoid bias on gender. Yellow indicates amplification and blue indicates deletion. The three groups are shown with the black horizontal lines in the heatmap. Group 1 is characterized by large area of deletion in chromosome 8. Group 2 has amplification in various regions of chromosome 2 to 6 and deletion in chromosome 17 and 18. Group 3 appears to have sparse aberrations in random regions.

**Figure 6:**
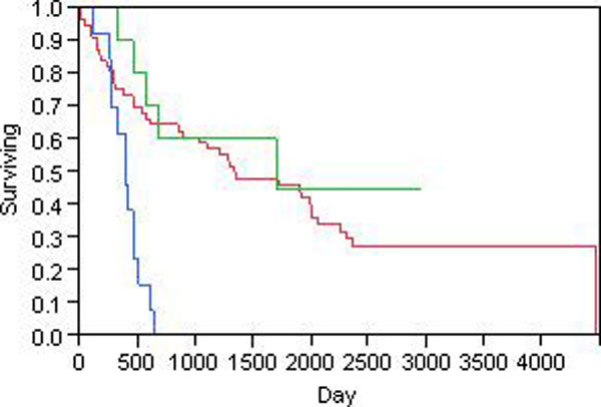
**Kaplan-Meier curves of glioma clusters**. The 79 glioma patients were clustered into 3 groups using HMMC. The Kaplan-Meier curves for overall survival time in days were shown. The blue line is group 1, green is group 2, and red is group 3. The P value by log-rank test is less than 0.0001.

To verify the clustering results, we performed survival analysis to the 3 groups. The Kaplan-Meier curves are shown in Figure 6. The blue line is group 1, the green is group 2 and the red is group 3. The median survival time for group 1 is 395 days, whereas those for group 2 and 3 are 1,698 and 1,337 days, respectively. Group 1 has a significant lower overall survival than group 2 and 3. The overall P value by log-rank test is less than 0.0001. Therefore, HMMC successfully clustered glioma samples based on aCGH data and the resulting tumor subtypes were associated with clinical outcomes. As discussed previously, group 2 manifests with large segments of deletion in chromosome 8. Thus genomic signature is identified for poor clinical outcome and they can serve as prognostic biomarkers. Upon identification of genes located in these abnormal genomics regions, we can discover the potential target for cancer therapy.

## Discussion

Genomic information has been increasingly used for molecular classifications of tumors because it provides a more objective view than histopathological approach and it sheds light on the molecular mechanisms of tumor heterogeneity. The global gene expression of major tumor types has been extensively studied for subtype identification [24-27]. The fast development of SNP array has made it possible to characterize tumor subtypes based on the genomic DNA copy number, nonetheless, the computational and statistical methods in this area is still under-developed. In many aspects, DNA copy number is better than RNA expression data in terms of genomic signature and diagnostic or prognostic biomarkers. DNA is much easier to store than RNA because DNA is a stable molecule whereas RNA is transient and easy to degrade. In addition, RNA has to be collected from fresh tissues whereas DNA can be isolated from frozen or paraform tissues even after years of storage. Furthermore, DNA aberration is preserved in the cell and can be passed to daughter cells via mitosis, whereas RNA expression is unstable and RNA levels are affected by many factors such as cell cycle, environmental and physiological factors. Therefore, tumor classification and genomic signature identification based on DNA copy number have extensive applications and improvements in the computational and statistical methods are needed to extend research in aCGH data analysis.

Most current statistical and data mining methods for aCGH data were developed from expression microarray analysis. An important difference between aCGH data and mRNA expression data is the high spatial correlation between neighboring SNPs in the aCGH data. The correlation between genes in expression microarray is relatively less and ignored by most methods that have been developed for such data. Thus most methods developed for expression microarray may yield significant false positives and false negatives when applied to the aCGH data. Here we proposed a clustering algorithm that relies on HMM to take into account of the correlations between aCGH markers (SNPs). We found our HMMC algorithm to perform better than the NMF clustering method, which is one of the most widely used methods used for aCGH sample clustering.

Though HMM has been widely used in modelling correlated data, such as DNA copy number data, it is widely known to be computationally very slow, especially for the analysis of high dimensional data such as the aCGH data. In order to overcome this problem, we have used various approaches to increase the computational efficiency and speed. First, in the clustering procedure, we conduct a preliminary NMF clustering and the resulting groups are used as the starting point for HMMC.

Secondly, instead of performing exhausting search, we only disturb the current optimal clustering with a few random labelling. Thirdly, we implement the algorithm in C++ that is much faster than R and MatLab. Furthermore, we have developed a parallel computing version of the program, and it increases the speed by over 10-fold our server computer. Our testing on 79 glioma samples showed that HMMC rapidly converges to its optimal clustering and the computation time is linear to the sample size.

## Conclusions

In this manuscript we proposed a HMM-based clustering algorithm for identifying tumor subtypes using aCGH DNA copy number data. This approach properly models the high spatial correlation between aCGH markers. Clusters of tumor samples are modelled with a mixture of HMM models where each HMM fits a cluster of samples. We have developed a computationally efficient and fast clustering algorithm that takes only a computational time of *O*(n). We have shown that this HMMC algorithm has less than half the error rate of NMF clustering and it can locate the optimal number of groups automatically while applying to glioma aCGH data. The resulting clustering of glioma samples has strong association with overall survival time. This HMMC algorithm would potentially have wide applications in tumor subtype identification, genomic signature discovery, and diagnostic and prognostic biomarker search. We will conduct future research in extending HMM-based modelling to other high dimensional biological data analysis, such as copy number variation, gene set enrichment analysis, and sequencing data analysis such as ChIP-seq data processing.

## Competing interests

The authors declare that they have no competing interests.

## Authors' contributions

KZ conceived the study, developed the HMMC algorithm and implemented it in R environment. YY wrote the C++ code for the methodology and performed test for glioma data. VD and YD provided constructive suggestions. KZ, VD, LX and DS wrote the manuscript with input from all authors. All authors read and approved the final manuscript.
